# Supplementary material for: Reconciling Mining with the Conservation of Cave Biodiversity: A Quantitative Baseline to Help Establish Conservation Priorities
Source: PLoS One. 2016 Dec 20;11(12):e0168348. doi: 10.1371/journal.pone.0168348 (PMC5173368; doi:10.1371/journal.pone.0168348)
Supplement: S1 Dataset — (ZIP) [file pone.0168348.s002.zip › Taxa/Serra Norte/SN_2013/N4E/Lista N4E-34.pdf]

CAVIDADE N4E-0034

Classe	Ordem	Fam/Outros	Gên/Outros	Espécie	S-U	U-S	Zon.
Arachnida	Acari	Anystidae	*Erythracarus*	sp.1	X		P
Arachnida	Amblypygi	Phrynidae	*Heterophrynus*	*longicornis*	1		E
Arachnida	Araneae	Corinnidae		jovem	1		E
Arachnida	Araneae	Nesticidae		jovem	X		E
Arachnida	Araneae	Pholcidae	*Leptopholcus*	sp.1	X		
Arachnida	Araneae	Pholcidae	*Mesabolivar*	sp.1	X	X	
Arachnida	Araneae	Theridiidae		jovem	X		E
Arachnida	Araneae	Theridiosomatidae	*Plato*	sp.		X	
Arachnida	Opiliones	Cosmetidae	*Roquettea*	*singularis*	2		
Arachnida	Opiliones	Stygnidae	*Protimesius*	*gracilis*	1		
Arachnida	Pseudoscorpiones	Chernetidae	*Spelaeochernes*	sp.1	X		E/P
Arachnida	Pseudoscorpiones	Chthoniidae		jovem	X		E
Chilopoda	Scolopendromorpha	Scolopendridae	*Otostigmus*	sp.1	1		
Entognatha	Collembola	Entomobryidae		sp.1	X		E
Entognatha	Collembola	Paronellidae		sp.7	X		P
Entognatha	Collembola			sp.		X	
Entognatha	Diplura	Campodeidae		sp.1	X		E
Insecta	Blattodea			jovem	1		E
Insecta	Coleoptera	Erotylidae		sp.1	X		P
Insecta	Coleoptera	Scydmaenidae		sp.1	X		E
Insecta	Diptera	Chironomidae		sp.	X		E/P
Insecta	Diptera	Psychodidae	Phlebotominae	sp.	X		E
Insecta	Diptera	Tipulidae	Tipulinae	sp.	X	X	
Insecta	Hymenoptera	Formicidae	*Camponotus*	sp.1	X	X	E/P
Insecta	Hymenoptera	Formicidae	*Paratrechina*	sp.1	X		E/P
Insecta	Hymenoptera	Formicidae	*Solenopsis*	sp.1	X		P
Insecta	Isoptera	Termitidae	*Nasutitermes*	sp.	X		P
Insecta	Orthoptera	Phalangopsidae	*Paraclodes*	sp.	2		E
Insecta	Orthoptera	Phalangopsidae	*Phalangopsis*	sp.	10	X	E/P
Insecta	Psocoptera			jovem	X		E
Insecta	Thysanura	Nicoletiidae		sp.1	X		P
Gastropoda	Pulmonata	Bulimulidae	*Naesiotus*	sp.	X		P
Gastropoda	Pulmonata	Subulinidae	*Lamellaxis*	sp.		X	
Gastropoda	Pulmonata	Subulinidae	*Leptinaria*	sp.		X	
Gastropoda	Pulmonata	Systrophiidae	*Happia*	sp.	X		E/P
Malacostraca	Isopoda	Scleropactidae	*Circoniscus*	*buckupi*		X	
Amphibia	Anura	Strabomantidae	*Pristimantis*	cf. *fenestratus*	3		E/P
Mammalia	Chiroptera	Emballonuridae	*Peropteryx*	*kappleri*	1		E
